# Type 1 Interleukin-4 Signaling Obliterates Mouse Astroglia *in vivo* but Not *in vitro*

**DOI:** 10.3389/fcell.2020.00114

**Published:** 2020-02-26

**Authors:** Violeta Mashkaryan, Tohid Siddiqui, Stanislava Popova, Mehmet Ilyas Cosacak, Prabesh Bhattarai, Kerstin Brandt, Nambirajan Govindarajan, Andreas Petzold, Susanne Reinhardt, Andreas Dahl, Roger Lefort, Caghan Kizil

**Affiliations:** ^1^German Center for Neurodegenerative Diseases Dresden, Helmholtz Association, Dresden, Germany; ^2^Department of Pathology and Cell Biology, Columbia University Irving Medical Center, New York, NY, United States; ^3^DRESDEN-Concept Genome Center, Center for Molecular and Cellular Bioengineering, TU Dresden, Dresden, Germany; ^4^Center for Regenerative Therapies Dresden, Center for Molecular and Cellular Bioengineering, TU Dresden, Dresden, Germany

**Keywords:** interleukin-4, STAT6, astroglia, mouse, Alzheimer’s disease, neurogenesis, regeneration, zebrafish

## Abstract

Recent findings suggest that reduced neurogenesis could be one of the underlying reasons for the exacerbated neuropathology in humans, thus restoring the neural stem cell proliferation and neurogenesis could help to circumvent some pathological aspects of Alzheimer’s disease. We recently identified Interleukin-4/STAT6 signaling as a neuron–glia crosstalk mechanism that enables glial proliferation and neurogenesis in adult zebrafish brain and 3D cultures of human astroglia, which manifest neurogenic properties. In this study, by using single cell sequencing in the APP/PS1dE9 mouse model of AD, we found that IL4 receptor (*Il4r*) is not expressed in mouse astroglia and IL4 signaling is not active in these cells. We tested whether activating IL4/STAT6 signaling would enhance cell proliferation and neurogenesis in healthy and disease conditions. Lentivirus-mediated expression of IL4R or constitutively active STAT6VT impaired the survival capacity of mouse astroglia *in vivo* but not *in vitro*. These results suggest that the adult mouse brain generates a non-permissive environment that dictates a negative effect of IL4 signaling on astroglial survival and neurogenic properties in contrast to zebrafish brains and *in vitro* mammalian cell cultures. Our findings that IL4R signaling in dentate gyrus (DG) of adult mouse brain impinges on the survival of DG cells implicate an evolutionary mechanism that might underlie the loss of neuroregenerative ability of the brain, which might be utilized for basic and clinical aspects for neurodegenerative diseases.

## Introduction

Alzheimer’s disease (AD) is a progressive and yet irreversible neurodegenerative disease. It is characterized by a progressive loss of neurons due to the Amyloid-mediated neurotoxicity that leads to a memory loss, cognitive decline, and eventually inability to perform simple tasks (Beyreuther and Masters, 1997; Selkoe, 2001, 2002, 2003; Brown et al., 2005; Blennow et al., 2006; Harman, 2006). Alzheimer’s pathology manifests due to malfunctioning of several cell types including neurons, immune cells, neurovascular compartment and astroglia (De Strooper and Karran, 2016; Scheltens et al., 2016). The pathology in the neural compartments leads to loss of synaptic connections and eventually to neuronal death while immune cells cause a chronic inflammatory environment and exacerbate neuronal loss (Heneka et al., 2015; Heppner et al., 2015; Jay et al., 2015; Liddelow et al., 2017). Modulation of inflammatory environment and efforts to retain the synaptic integrity during the course of AD are promising approaches to revert the neuropathological changes of the disease, yet other cellular paradigms such as neurogenesis could be involved in manifestation of AD phenotypes (Amor et al., 2010; Rodriguez and Verkhratsky, 2011; Heneka et al., 2013; Nisbet et al., 2015; De Strooper and Karran, 2016; Dzamba et al., 2016; Scheltens et al., 2016; Kizil, 2018). Recently, several studies suggested that in AD patients neurogenesis is significantly reduced compared to healthy individuals (Rodriguez et al., 2008; Rodriguez and Verkhratsky, 2011; Tincer et al., 2016; Kizil and Bhattarai, 2018; Choi and Tanzi, 2019; Moreno-Jimenez et al., 2019). This might indicate that impaired neurogenesis, a physiological phenomenon that has not been investigated extensively in AD, could be a factor in the manifestation of AD pathology (Cosacak et al., 2015; Tincer et al., 2016; Kizil, 2018; Kizil and Bhattarai, 2018; Choi and Tanzi, 2019; Cosacak et al., 2020). Indeed, one of the early symptoms of AD in mouse models is reduced neural stem cell proliferation and neurogenesis (Haughey et al., 2002a, b; Ziabreva et al., 2006), and increased neurogenesis – when experimentally coupled to neuronal survival in AD mouse brains – can revert the cognitive decline (Choi et al., 2018). These findings suggest that enhancing neurogenesis might be a way to counteract AD progression by “regenerating” neurons. However, our knowledge of the molecular mechanisms by which neural stem cells could enhance their proliferation and neurogenic ability in disease conditions is limited.

We identified that in a zebrafish model of AD, Amyloid-mediated pathology induces neural stem cell proliferation and subsequent neurogenesis and integration of newborn neurons into the brain despite the prevalent neurodegenerative toxicity (Bhattarai et al., 2016, 2017a,b, 2020; Cosacak et al., 2019). Our findings suggested that IL4 could be a mechanism to enable neural stem cell plasticity and neurogenesis in AD conditions. To test this hypothesis, we generated a 3D hydrogel culture model where astroglia from fetal human cortex or iPSC-derived neural stem cells were encapsulated and exposed to aggregated amyloid (Papadimitriou et al., 2018; Celikkaya et al., 2019). We found that IL4/STAT6 signaling could revert the reduced proliferative and neurogenic ability of human astroglia upon Amyloid toxicity in 3D hydrogels *in vitro*. Based on these results, we hypothesized that IL4/STAT6 signaling could also enhance neurogenesis in mouse brains *in vivo* during health and in AD.

**FIGURE 1:**
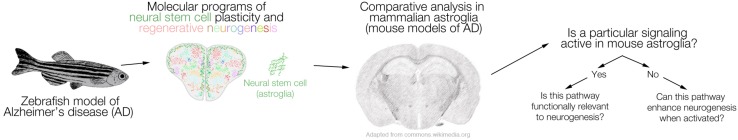
A schematic representation of the comparative logic between zebrafish and mammalian models of Alzheimer’s disease (AD). The regenerative neurogenic ability of zebrafish brain can be harnessed to elucidate the mechanisms, which can be further tested in mouse models of AD. If a particular signaling that enables regeneration in zebrafish brain is active also in mouse brains (e.g., in neural stem cells or progenitors), functional relevance to neurogenesis can be tested. If such a program ceases to exist in mammalian brains, activation of the pathway or its components can be tested as to whether they induce the regenerative neurogenic ability in mammalian brains. We propose this workflow as a stringent comparative analysis pipeline between zebrafish and mammalian brains.

We conceptualized that if a molecular program is active in zebrafish astroglia and this program is involved in regenerative neurogenesis, it would be interesting to see whether this program is also active in mammalian astroglia *in vivo*, and whether it has a similar role (e.g., regenerative neurogenesis). In our previous studies, we found that a toxicity-specific neuron–glia interaction through Interleukin-4 (*il4*) that is expressed by immune cells and dying neurons, and its receptor *il4r*, which is specifically expressed in the neural stem cells with radial glial identity enable toxicity-induced neurogenesis through STAT6 signaling (Bhattarai et al., 2016, 2020; Cosacak et al., 2019). This naturally led us to investigate whether mouse astroglia expressed *Il4r*, and if not, whether the astroglial proliferation and neurogenesis would enhance after activating the signaling *in vivo*. This approach can be generalized as a workflow for future studies (Figure 1).

IL4 is an anti-inflammatory chemokine that plays a key and complex role in polarization of the microglia and resolution of the inflammation (Rolling et al., 1996; Chen et al., 2003; Kelly-Welch et al., 2003; Lyons et al., 2007). After the onset of inflammation- for instance in disease states – IL4 downregulates pro-inflammatory cytokines TNF and IL1β (Hart et al., 1989). Such a relief of inflammatory environment was suggested to ameliorate the disease-associated outcomes such as pro-inflammatory milieu, neuronal death, or excitotoxicity (Suzumura et al., 1994; Garg et al., 2009) and act as a neuroprotective mechanism. In neurons, long-term potentiation is enhanced after IL4 during aging and AD conditions in rodents *in vivo* (Maher et al., 2005; Kiyota et al., 2010). In mouse AD and amyloidosis models, the role of IL4 is controversial. Synaptic degeneration alleviates when key inflammasome component NLRP3 is knocked-out in mice and these mice increase the expression of *Il4*. However, the increase in synaptic integrity is possibly not a direct consequence of the enhanced *Il4* expression but rather the microglial dynamics (Heneka et al., 2013). Overall, IL4 has a beneficial role on the homeostatic functions of the brain and it ameliorates AD symptoms by suppressing the inflammation and producing a permissive environment (Maher et al., 2005; Nolan et al., 2005; Lyons et al., 2007, 2009; Clarke et al., 2008; Gadani et al., 2012; Barrett et al., 2015).

The effect of IL4 on the proliferative potential and neurogenic ability of astroglia is unclear. According to one study a viral mediated overexpression of murine IL4 in the APP/PS1 mouse model of AD leads to a reduction of amyloid induced gliosis and amyloid peptide deposition together with improvement of neurogenesis (Kiyota et al., 2010). Yet, the worsening of AD-like symptoms upon overexpression of murine IL4 was also proposed by another study that used another mouse model for AD – TgCRND8 (Chakrabarty et al., 2012). So far, IL4 signaling was not investigated specifically in astroglia and the studies addressing the changes in neurogenesis after IL4 peptide injection into the mouse brain resulted in varying outcomes due to its direct effects on the immune environment and microglia. In our zebrafish Amyloid toxicity model, microglia is activated rapidly concomitant to the upregulation of *il4* expression, the prevalence of which overlaps with the neurogenic burst and morphological changes in the microglia (Bhattarai et al., 2016). We believe that determining the cell types expressing Interleukin-4 receptor (*Il4r*) would provide a further understanding on the confounding roles of IL4 in the complex milieu of the mouse brain. Additionally, enhancing IL4 signaling in astrocytes would help addressing any cell autonomous effects of this signaling pathway. Finally, investigating the neuroregenerative response in a complex mammalian brain system would help generating models that could better resemble the human brains and could contribute to designing clinical avenues for neuroregenerative therapies.

Therefore, in our study, we aimed to determine (1) whether IL4R is expressed in mouse astroglia by performing single cell sequencing and immunohistochemical stainings, and (2) whether active IL4R signaling could affect the proliferative and neurogenic ability of astroglial cells in mouse brains. Since astrocytes are the primary sources of new neurons by acting as neural stem cells in special niches (Doetsch et al., 1999; Doetsch and Scharff, 2001; Alvarez-Buylla et al., 2002; Doetsch, 2003a, b), investigating the effects of certain signaling pathways in these cell types hold the promise for a yet-elusive “induced regeneration” response of the mammalian brains.

## Materials and Methods

### Ethics Statement

All animal experimental procedures were approved by Landesdirektion Sachsen, under license number TVV 87/2016, and followed the safety regulations of DZNE Dresden and TU Dresden. All precautions were taken to minimize animal suffering and to reduce animal numbers. Wild type and age-matched APP/PS1dE9 (Janus et al., 2015) animals were used for this study.

### Lentiviral Construct Production

The generation of HIV-1 pseudo-typed virus was achieved by a co-transfection of three plasmids in HEK293T cells: (I) pCD/NL-BH – packaging plasmid that contains the Gag, Pol, Rev, and Tat genes; (II) pczVSV-Gwt – envelope plasmid that encodes the VSV-G protein; and (III) p6NST90 – transfer vector plasmid (Dirk Lindemann, University Clinic Dresden, Germany) that contained the genes of interest: the IL4R or STAT6VT. The sequences for the full length of hIL4R and hSTAT6VT together with T2A were cloned into the backbone of the HIV transfer vector using the following primers: hIl4r_FW 5′–3′; hIl4r_RV 5′-accggtaaagggccgggattctcctcca-3′; hIl4r_NLS_FW 5′-ACCGGTgcggccatgggtctcacctcccaactgctt-3′; hIl4r_NLS_RV 5′-CACGTCACCGCATGTTAGAAGACTTCCTCTGCCCTCgctcg aacactttgaatatttct-3′; Stat6VT_FW 5′-accggtgccaccatgtctctgtgg ggtctggtct-3′; Stat6VT _RV 5′-accggtaaagggccgggattctcctccacgtc accgcatgttagaagacttcctctgccctcccaactggggttggccct-3′. The plasmid for hSTAT6VT was a gift from Mark H Kaplan (Indiana University) (Kaplan et al., 2007; Sehra et al., 2010).

The generation of p6NST90-based replication-deficient lentivirus particles and transduction of target cells were based on a permit by the Sächsisches Staatsministerium für Umwelt und Landwirtschaft (Az. 54-8452/78/10). For transfection, 5 million HEK293T cells were seeded in a 10 cm dish in 8 ml of DMEM (10% heat-inactivated FBS, 1% Pen/Strep). For one virus preparation, we used 18–21 dishes. After 24 h post-seeding, for every dish 1 ml of pre-warmed blank DMEM without FBS and Pen/Strep was mixed with 5 μg of each of the three plasmids (pCD/NL-BH, pczVSV-Gwt and p6NST90 with cloned transgenes). Next, 45 μl of polyethylenimine (PEI, 1 mg/ml) were diluted in 1 ml of blank DMEM per dish. The PEI solution was added rapidly to the plasmid solution, and incubated for 30 min at room temperature. Fresh DMEM with 15% heat-inactivated serum and 1% Pen/Strep were added to each dish (4 ml/dish) and the transfection mixture was added on top. At 30 h post-transfection, media was changed by adding 5 ml DMEM (1% Pen/Strep, no FBS) to each dish.

At 48 h post-transfection the supernatants were collected, filtered and concentrated by ultracentrifugation. The generated viral pellets were then re-suspended in PBS as documented before (Stirnnagel et al., 2010; Ho et al., 2012). The presence of viral particles was tested using Lenti-X^TM^ GoStix^TM^ Plus (Takara Cat.-No. 631280) and the presence of GFP expression in transfected or transduced target cells was verified by fluorescent microscopy.

### Culture of Adult Mouse Neural Stem/Progenitor Cells (NSPCs)

Adult neural stem/progenitor cells were isolated from the dentate gyri (DG) of 3 month-old WT mice following an optimized version of an established protocol (Hagihara et al., 2009; Walker et al., 2009; Walker and Kempermann, 2014). Mice were sacrificed by cervical dislocation. DG from both hemispheres were microscopically dissected on ice in PBS containing Pen/Strep. Tissues were then further minced using a scalpel and transferred to 1.5 mL tubes for dissociation using Neural Tissue Dissociation Kit from Miltenyi Biotec. The dissociated single cell suspensions were plated in a PDL/Laminin coated 25 cm^2^ culture flask and incubated at 37°C with 5% CO_2_. Cells were expanded and passaged as monolayers in a complete Neurobasal Media. Media was exchanged every 48 h. Only passages 8–12 were used during the experiments.

### Stereotaxic Injections of Lentiviral Vectors and Astroglia

The viral injections into wild type mouse brains were carried out in an S2-approved laboratory. All the regulated precautions were met to prevent the direct contact of personnel with viruses and to avoid infecting the animal during the operation. The procedure was carried out according to previously established protocol (Artegiani et al., 2011). During the entire surgery the mice were anesthetized using a mix of oxygen and isoflurane (49:1) (Baxter – HDG9623) flow and placed on a pre-warmed heat-pad to prevent hypothermia. 1 μl of the respective virus was injected at the coordinates: (a) ± 1.6 mm mediolateral, −1.9 mm anterior–posterior and −1.9 mm dorsoventral from the Bregma for the hippocampus at 200 nl/min speed; and (b) ± 1.0 mm mediolateral, −1.0 mm anterior–posterior and −0.8 mm dorsoventral from pia for the cortex at 50 nl/min speed. Contralateral hemispheres of wild type animals were used for injecting Lv-UbiC:GFP or Lv-UbiC:IL4R-GFP viruses. Brains were isolated at 2 weeks post-injection after the last BrdU injection. BrdU was administered intraperitoneally in the concentration of 50 mg/kg of body weight three times 6 h apart. The transplantation procedure is an independent experiment and technically was performed as described for the virus injection.

For the transduction of astroglia before transplantation, cells were seeded in a 24-well plate coated with PDL (100 μg/ml) and Laminin (0.01% w/v). After 48 h cells reach 70–80% confluency. The respective virus was then added to each well (10^9^ infection units). Cells were incubated with the virus for 24 h at 37°C with 5% CO_2_, after which the media was exchanged and cells were allowed to grow till 90% confluency. At this point cells were ready for either transplantation. Transduced cells were trypsinized immediately before transplantation to avoid keeping cells on ice for longer than 1 h. 1.5 μl of 1 × 10^5^ cells/μl suspension was manually delivered to each hemisphere at 100 nl/min speed. Virus titers were 10^9^ infection units per milliliter for injection into the mouse brain and transduction of cells *in vitro*. Mice were sacrificed 1 week after injection.

### Immunohistochemistry

Mice were anesthetized with an intraperitoneal injection of a mixture of Ketamine (100 mg/kg) and Xylazine (10 mg/kg) and then transcardially perfused with NaCl 0.9% followed by cold freshly prepared 4% PFA. Brains were further post-fixed in 4% PFA overnight at 4°C. 40 μm-thick free-floating sections were made on a microtome and collected in six consecutive series in a cryo-preservation solution [0.1M Phosphate buffer, 25% (v/v) ethylene glycol, 25% (v/v) glycerol]. One serial group of free-floating sections were washed in PBS, blocked in PBS + (10% donkey or goat serum, 0.2% TritonX, 1x PBS) for 1 h at RT and incubated overnight at 4°C with the desired primary antibody of defined dilution in PBS + (3% donkey or goat serum, 0.2% Triton-X, 1x PBS). Sections were washed three times within 1 h and incubated for another hour at RT with the respective secondary antibody (1:500) coupled to a desired fluorophore. After short while, wash samples were then incubated in DAPI diluted in PBS (1:5000) for 10 min. Another series of washes were done and samples were mounted on the charged glass slides. After mounting, slides were left to dry and covered with a coverslip using Aqua Mount.

The fixed cell cultures were permeabilized with 0.1% Triton-X in PBS for 5 min at RT followed by 10 min blocking with 5% goat or donkey serum and 0.1% Triton-X in PBS at RT. Cells were then washed with PBS and primary antibodies of the required dilutions in PBS were added. Cells were incubated for 1 h at RT, washed three times for 5 min with PBS. Secondary antibodies diluted in PBS (1:500) were added and cells were incubated for another hour at RT followed by DAPI treatment for 10 min (1:5000 in PBS). At the end cells were washed three times for 5 min in PBS. At this point cells were either kept in PBS in 4°C or proceeded to imaging.

Samples were imaged on a ZEISS fluorescent microscope with ApoTome using 10x/0.45 20x/0.4 40x/0.95 objectives. Images were acquired using ZEN software and analyzed using ZEN and FIJI software (version 2.0.0.).

### Single-Cell Sequencing

The DGs from WT and APP/PS1 mice were dissected in ice cold PBS with Pen/Strep and the cell dissociation was done using Neural Tissue Dissociation Kit (P) (Miltenyi Biotech) as described (Bhattarai et al., 2016). Cells were sorted by (BD FACS Calibur^TM^) flow cytometry using Propidium Iodide cell viability dye to exclude dead cells. Subsequently, alive cells were directly loaded onto a 10x A-chip after mixing them with reverse transcriptase master mix. GEM generation, cDNA synthesis and amplification (for eight cycles) as well as library preparation was performed with Chromium Next GEM Single Cell 3′ GEM, Library & Gel Bead Kit v2 (10x Genomics) according to the manufacturer’s protocol (Zheng et al., 2016). Read alignment and read counts were done by Cell Ranger 2.1.0. For data analysis Seurat R package (Butler et al., 2018; Farrell et al., 2018) was used as described in Cosacak et al. (2019). In total, two replicates from the same cell mix were processed and sequenced. All count matrices were imported by Read10X function of Seurat and uniquely named to trace back cells if required. In a first step cells with either more than 10000 UMI and less than 1000 UMI, or less than 500 and more than 2500 unique genes were filtered out, likewise cells with more than 6% mitochondrial genes. Further, genes found in less than 10 cells were excluded. The remaining cells and genes were used for downstream analysis for all samples. The data was normalized using the “LogNormalize” method, data scaled with “scale.factor = 1e4,” nUMI, nGene and batch effects were regressed out. For each datasets variable genes were found with FindVariableGenes with the following options mean.function = ExpMean, dispersion.function = LogVMR, x.low.cutoff = 0.125, x.high.cutoff = 10, y.cutoff = 0.5. The top 1000 most variable genes from every sample (determined by Seurat) were merged. Then, the intersection of these genes with all genes in each samples were used for CCA analysis. The two Seurat objects and the variable genes found above were used to generate a new Seurat object with RunCCA function, using num.ccs = 30. The canonical correlation strength was calculated using num.dims = 1:30 and the samples were aligned using dims.align = 1:20. The cell clusters were found using aligned CCA and 1:10 dims, with resolution 0.5. Each cell cluster named based on the markers. 1,324 cells from wild type and 1,429 cells from APP/PS1dE9 mouse hippocampi were analyzed. The raw data BAM files and matrices can be found in GEO^1^ (accession number: GSE140793). All R scripts are available on kizillab.org/resources.

### List of Antibodies Used


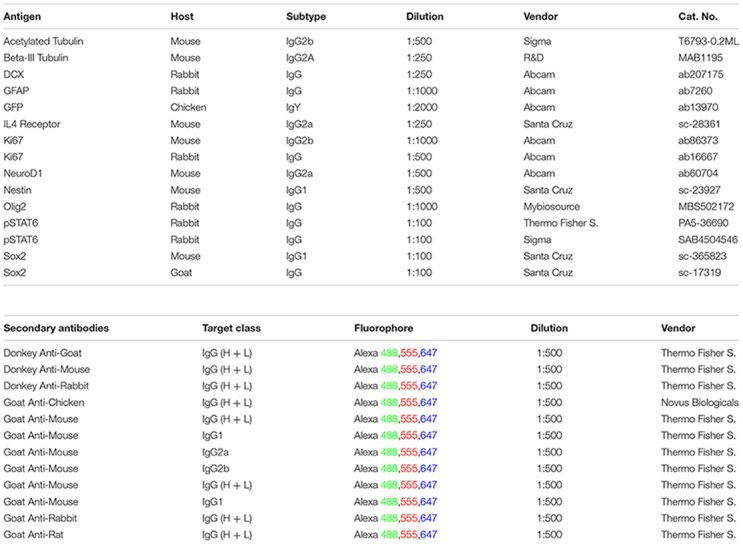


### Animal Maintenance

Mice were housed in a 12 h alternating light/dark cycle with food and water *ad libitum*. All animal experimental procedures were approved by local authorities, and all reasonable precautions were taken to minimize animal suffering and to reduce animal numbers. BrdU (Bromodeoxyuridine) was administered intraperitoneally in concentration of 50 mg/kg of body weight three times with 6 h intervals. Depending on the question, animals were sacrificed from 12 h to 2 weeks after the third injection. Animals were sacrificed by Ketamin/Xylazine mixture and perfused using filtered 0.9% saline solution followed by a 4% PFA.

### Quantification and Statistical Analyses

The cells that were positive for BrdU were only counted when they appeared in the two-cell layer thick area adjacent to the SGZ of the DG. Cells were counted for 1/6th of the entire mouse brain and extrapolated for the whole brain. Five animals per condition were used. Student’s *t*-test were used with the significance level (α = 0.05). Graphs represent mean and standard deviation.

## Results

### Aβ42 Reduces the BrdU-Positive Cells in the Neurogenic Zone of the Dentate Gyrus and Increases Reactive Gliosis

**FIGURE 2:**
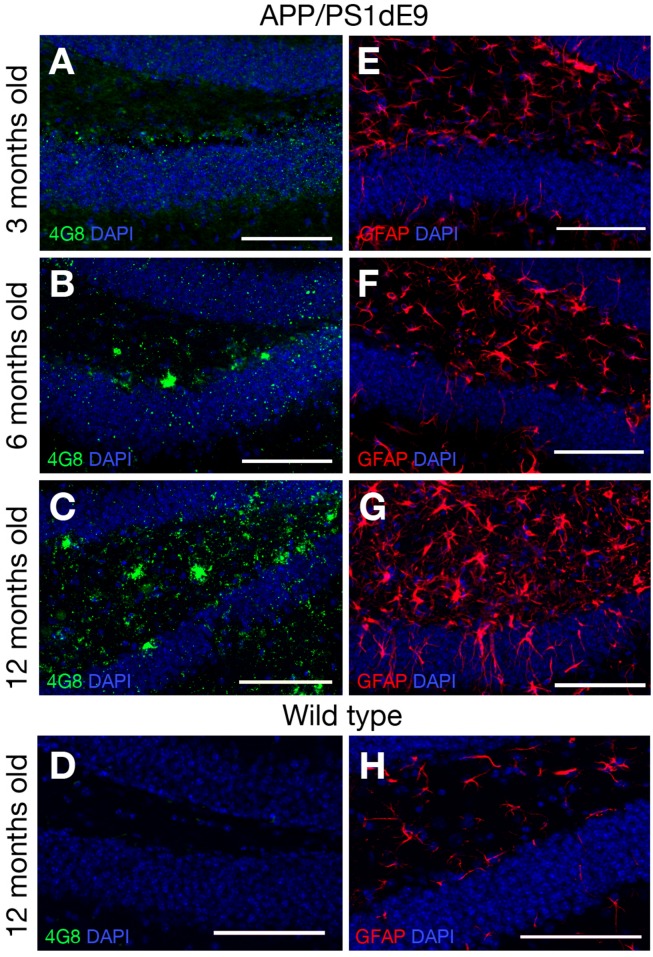
**(A–C)** 4G8 immunolabeling on brain sections of APP/PS1dE9 AD mouse model (3, 6, 12 months old respectively). **(D)** 4G8 staining on wild type mouse (12 months old). **(E–G)** GFAP immunolabeling on brain sections of APP/PS1dE9 mouse (3, 6, 12 months old respectively). **(H)** GFAP staining on wild type mouse (12 months old). *n* = 2 animals. Scale bars: 100 μm.

Transgenic AD mouse models display accumulation of amyloid and reactive gliosis (Chen et al., 2000; Donovan et al., 2006; Rodriguez et al., 2008; Lithner et al., 2011; van Tijn et al., 2011; Janus et al., 2015). To confirm these findings, we determined the accumulation of amyloid by performing immunolabelling for 4G8 – a widely used antibody that detects the amino acid residues 18–23 in Aβ peptides in abnormally processed isoforms as well as precursor forms – in 3, 6, and 12-month-old mice (WT and APP/PS1dE9) (Figure 2). In 3-month-old mice there was no immunoreactivity against 4G8 detected in the hippocampus. At 6 months of age, the first signs of 4G8-positive aggregations were observed and at 12 months the accumulation was widespread and abundant (Figures 2A–C). WT animals did not show any signs of plaques at 12 months of age (Figure 2D). To determine the level of reactive gliosis, the brains were immunolabeled against the GFAP that marks the astroglia. Compared to WT animals, the GFAP-positive activated astroglia with a distinctive morphology (increased in size as well as ramification and thickness of processes) were evident in the double transgenic animals as early as 3 months (Figures 2A,B,D). By 12 months a pronounced astrogliosis was observable that coincided with the plaque stage of Aβ42 (Figure 2C). These results confirm previous findings in this animal model (Donovan et al., 2006; He et al., 2013; Heneka et al., 2013; McClean and Holscher, 2014; Unger et al., 2016) and indicate that 12-month-old mice can be used to investigate the role of IL4/STAT6 signaling in the astroglia of diseased brain that manifests amyloid pathology.

**FIGURE 3:**
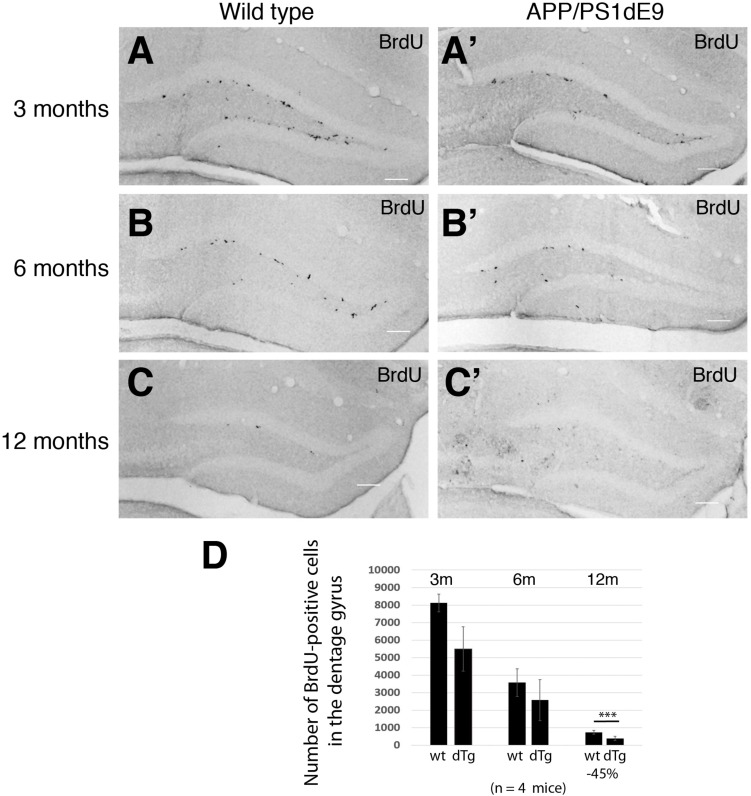
**(A–C)** BrdU immunostaining on cross sections of wild type mouse hippocampus at 3, 6, and 12 months of age. **(A′–C′)** BrdU immunostaining on cross sections of APP/PS1dE9 mouse hippocampus at 3, 6, and 12 months of age. **(D)** Quantification of total BrdU positive cells per mouse hippocampus. Proliferation reduces significantly at 12 months of age in APP/PS1dE9 mouse. *n* = 4 animals. Scale bars: 100 μm.

To determine how the proliferation of neural stem/progenitor cells change in APP/PS1dE9 animals as compared to controls, we performed BrdU pulses as described in Section “Materials and Methods,” performed BrdU immunolabeling stainings and stereologically quantified the proliferating cells at the stem cell niche of the hippocampus as described before (Kempermann et al., 2003). In WT animals, the levels of BrdU-positive proliferating neural stem/progenitor cells (NSPCs) declined with the age (Figure 3). The decline in the APP/PS1 animals however was more pronounced (Figure 3). The overall difference between the levels of proliferation in wild type and APP/PS1dE9 animals becomes statistically significant at 12 months where the strongest accumulation of Aβ and gliogenesis was observed (Figures 2, 3). This finding was also consistent with previous reports where NSPC proliferation reduces in AD mouse brains (Poirier et al., 2010; Mu and Gage, 2011; Tincer et al., 2016; Unger et al., 2016; Baglietto-Vargas et al., 2017; Choi et al., 2018; Teixeira et al., 2018; Choi and Tanzi, 2019).

### *Il4ra* Is Not Expressed in Mouse Astroglia

**FIGURE 4:**
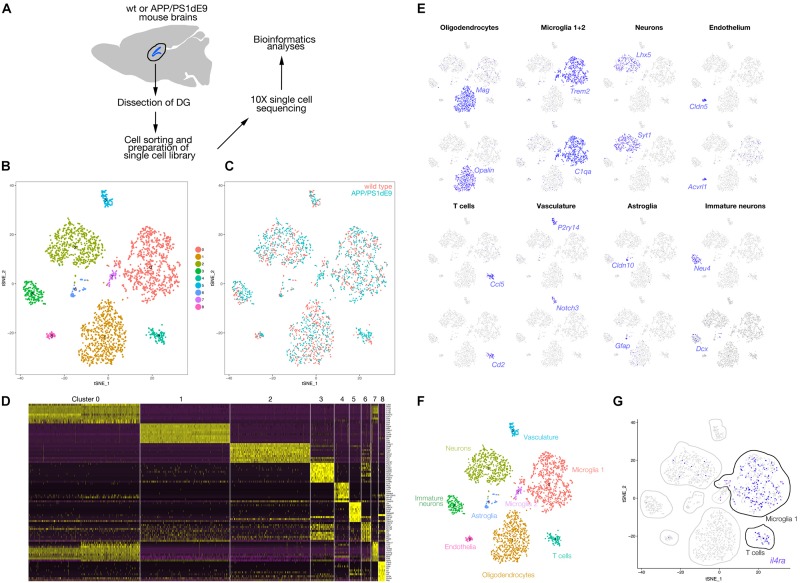
**(A)** Schematic overview of the single cell sequencing procedure. Dissected dentate gyri (DG) of hippocampi were dissociated into single cells, sorted by flow cytometry. Cells were subjected to 10X library preparation, followed by 3′ end transcriptome sequencing and bioinformatics analyses. **(B)** tSNE plot after cell clustering. **(C)** Cells from wild type and APP/PS1dE9 mouse DG plotted in different colors on the tSNE supporting a similar cell type composition and distribution. **(D)** Heat map for cell clusters showing top10 marker genes. **(E)** Feature plots for several genes known to be markers of various cell types. Cell clusters are named according to the marker gene expression: *Mag* and *Opalin* for oligodendrocytes, *C1qa* and *Trem2* for microglia, *Lhx5* and *Syt1* for neurons, *Cldn5* and *Acvrl1* for endothelial cells, *Ccl5* and *Cd2* for T cells, *P2ry14* and *Notch3* for vasculature, *Cldn10* and *Gfap* for astroglia, *Neu4* and *Dcx* for immature neurons. **(F)** Named cell clusters on tSNE plot. **(G)** Expression of *Il4r* on tSNE plot. IL4 receptor is expressed mainly in microglia and T cells. Very few neurons also express the receptor. *Il4r* is not expressed in astroglia.

**FIGURE 5:**
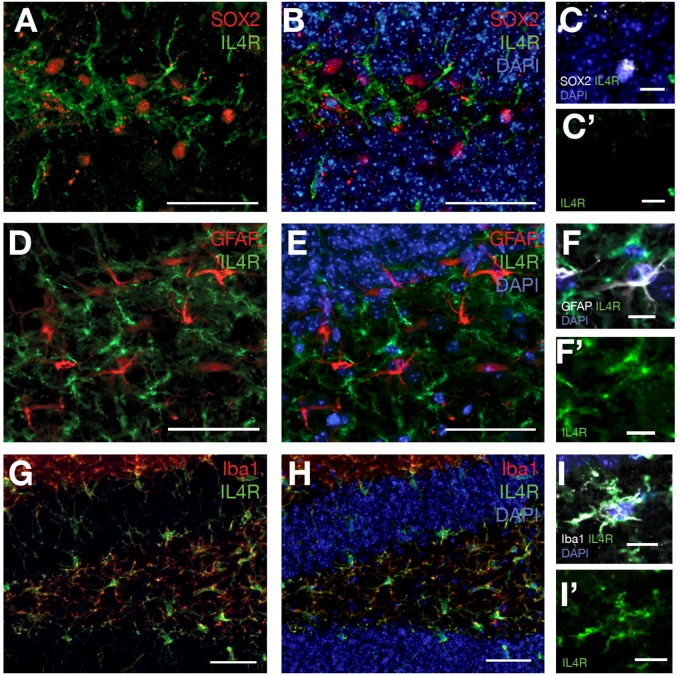
**(A)** SOX2 and IL4R immunostaining on brain sections of 12 months old wild type mouse. **(B)** DAPI added to **(A)**. **(C)** SOX2-positive cells do not express IL4R. **(C′)** IL4R fluorescence channel alone. **(D)** GFAP and IL4R immunostaining on brain sections of 12 months old wild type mouse. **(E)** DAPI added to **(D)**. **(F)** GFAP-positive cells do not express IL4R. **(F′)** IL4R fluorescence channel alone. **(G)** Iba1 and IL4R immunostaining on brain sections of 12 months old wild type mouse. **(H)** DAPI added to **(G)**. **(I)** Iba-positive cells express IL4R. **(I′)** IL4R fluorescence channel alone. *n* = 3 animals. Scale bars: 100 μm **(A,B,D,E,G,H)** and 25 μm elsewhere.

To identify the expression of Il4 receptor (*il4ra*) in the hippocampus, we performed single cell sequencing from wild type and APP/PS1dE9 mouse brains at 12 months of age (Figure 4). After clustering and identification of cell types (astroglia/NSPCs “AG/NSC,” oligodendrocytes “OD,” microglia “MG,” T-cells “TC,” pericytes/endothelial cells “PC/EC,” neurons “N,” Figure 4), we investigated the expression of *Il4ra* and found that only immune cells (MG, TC) that express the receptor while AG/NSCs are negative for *Il4ra* (Figure 4). To confirm our single cell sequencing results, we performed immunolabeling against IL4R in astroglia (GFAP-positive cells), in SOX2-positive cells, and in immune cells (Iba1-positive) (Figure 5). We indeed found that IL4R is expressed only in immune cells in mouse hippocampus. Based on these results, we hypothesized that if IL4R expression was induced in AG/NSCs, proliferation and neurogenesis could be enhanced similar to the zebrafish brain (Bhattarai et al., 2016) and *in vitro* in 3D human astroglia cultures (Papadimitriou et al., 2018).

### The Overexpression of IL4R in the Adult Mouse DG Using Viral Expression Vectors

**FIGURE 6:**
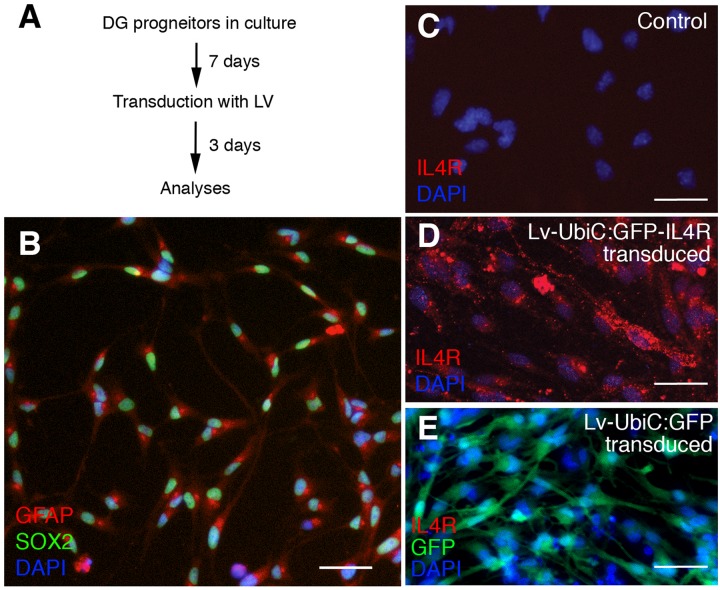
**(A)** Schematic view of cell culture and transduction of adult mouse dentate gyrus astroglia. **(B)** Immunostaining for GFAP and SOX2 in control cultures. **(C)** Immunostaining for IL4R in control cultures. **(D)** Immunostaining for IL4R after transduction with Lv-UbiC:IL4R-GFP. **(E)** Immunostaining for IL4R and GFP after transduction with Lv-UbiC:GFP. Scale bars: 25 μm.

To overexpress the IL4R in mouse astroglia, we generated lentivirus particles containing the human IL4R under the uniquitous promoter UbiC (LV-UbiC:IL4R-GFP) and used the empty GFP-expressing lentivirus backbone as control (LV-UbiC:GFP, Figure 6A and Supplementary Figure S1). To test the efficiency of transduction, we cultured adult mouse dentate gyrus progenitors (Figure 5A) that can form neurons *in vitro* after growth factor withdrawal (Supplementary Figure S2). These cells expressed GFAP and SOX2 (Figure 6B) but not IL4R (Figure 6C). Transduction with LV-UbiC:GFP-IL4R resulted in strong expression of IL4R and this did not change the viability of the cells (Figure 6D). LV-UbiC:GFP transduction displayed a similar efficiency of GFP expression (Figure 6E). We concluded that lentiviral particles expressing GFP or IL4R could transduce mouse astroglia and lead to the expression of the IL4R and GFP *in vitro*.

**FIGURE 7:**
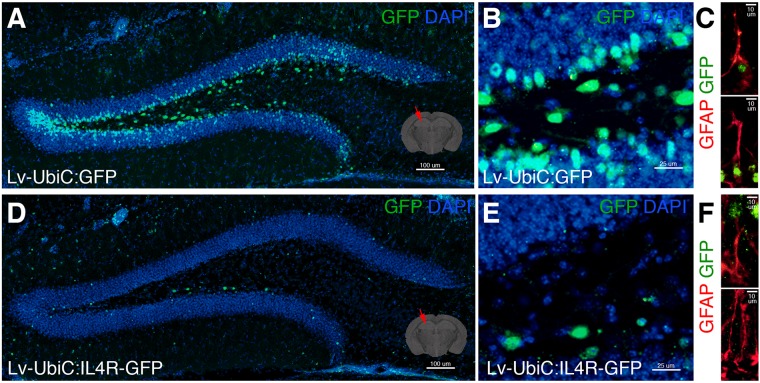
**(A)** GFP immunostaining on coronal section of wild type mouse brain after transduction of Lv-UbiC:GFP. **(B)** Close up image from **(A)**. **(C)** GFAP and GFP immunostaining showing Lv-UbiC:GFP-transduced astroglia. **(D)** GFP immunostaining on coronal section of wild type mouse brain after transduction of Lv-UbiC:IL4R-GFP. **(E)** Close up image from **(D)**. **(F)** GFAP and GFP immunostaining showing Lv-UbiC:IL4R-GFP-transduced astroglia. *n* = 3 wild type animals. Scale bars: 100 μm **(A,D)**, 25 μm **(B,E)**, and 10 μm **(C,F)**.

When we injected LV-UbiC:GFP into adult mouse dentate gyrus (Figure 7A), we observed transduction in the subgranular zone (SGZ) and we could target glial cells (Figures 7B,C). However, the injection of LV-UbiC:IL4R-GFP into the brains of wild type animals consistently resulted in a considerable lower number of transduced cells (Figures 7D,E) and we hardly saw any GFP-positive glia (Figure 7F). When we performed LV injection into the cortex, we observed that IL4R virus resulted always in lower number of transduced cells independent of the relative titers (data not shown). These results suggested that the astroglia expressing IL4R survive *in vitro* but not *in vivo*, proposing a non-permissive environment that impinges on the survival of the IL4R-expressing glia *in vivo*.

### Transplantation of Transduced Adult NSPCs

Since overexpression of IL4R via lentivirus injection impaired the survival of cells, we designed an alternative strategy to first transduce the mouse astroglia *in vitro* and then transplant these cells into the hippocampus or the cortex of the mouse brains. Regarding our experimental conditions, it could be possible that IL4R-expression after direct transduction by the virus injection might affect the astroglia because of the injection paradigm, the presence of the virus in the tissue. To eliminate these probabilities we used the transplantation paradigm. Additionally, we used an alternative strategy to express a constitutively active form of STAT6 (STAT6VT; Kaplan et al., 2007; Sehra et al., 2010) that would keep the IL4/STAT6 signaling continuously active. We specifically tried this because provided that the outcomes of IL4R expression and STAT6VT expression would be similar, we could be more confident about a specific effect of IL4R signaling on astroglia (by these two independent modes of activating the IL4R signaling).

**FIGURE 8:**
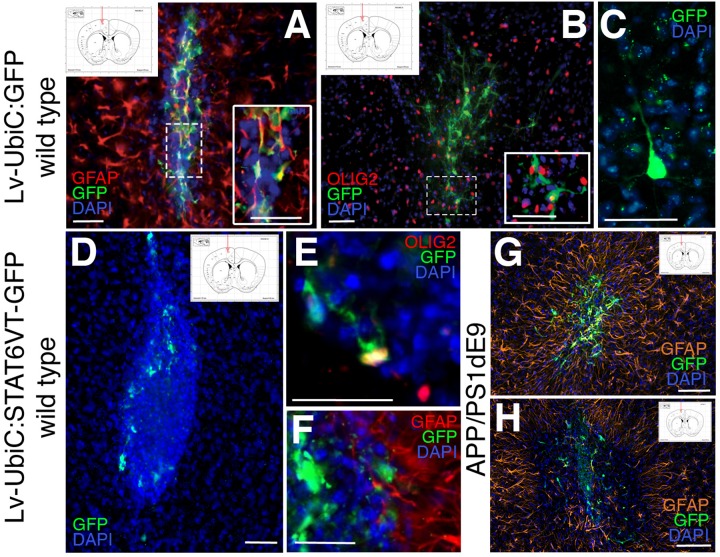
**(A)** Immunostaining for GFAP (red) and GFP after transplantation of astroglia transduced with Lv-UbiC:GFP into wild type mouse cortex. **(B)** Immunostaining for OLIG2 (red) and GFP after transplantation of astroglia transduced with Lv-UbiC:GFP into wild type mouse cortex. **(C)** Immunostaining for GFP after transplantation of astroglia transduced with Lv-UbiC:GFP into adult mouse cortex shows neuronal morphologies. **(D)** Immunostaining for GFP after transplantation of astroglia transduced with Lv-UbiC:STAT6VT-GFP into adult mouse cortex. **(E)** Immunostaining for OLIG2 (red) and GFP after transplantation of astroglia transduced with Lv-UbiC:STAT6VT-GFP into wild type mouse cortex. **(F)** Immunostaining for GFAP (red) and GFP after transplantation of astroglia transduced with Lv-UbiC:STAT6VT-GFP into wild type mouse cortex. **(G)** Immunostaining for GFAP (orange) and GFP after transplantation of astroglia transduced with Lv-UbiC:GFP into APP/PS1dE9 adult mouse cortex. **(H)** Immunostaining for GFAP (orange) and GFP after transplantation of astroglia transduced with Lv-UbiC:STAT6VT-GFP into APP/PS1dE9 adult mouse cortex. *n* = 3 animals. Schematic information on injection locations presented in the insets. Scale bars: 50 μm.

Mouse astroglia were transduced with the virus, collected at 2 days after transduction to allow the expression of the gene of interest (GFP and STAT6VT), transplanted into the hippocampal and the cortical region of 12-month old wild type mice and the brains were analyzed 1 week after the transplantation. Hippocampal transplantations did not yield any integration into the region (data not shown) but cortical transplantations did. Therefore, we continued with the analyses of the cortical transplantations. To determine which cell types are formed by transplanted transduced astroglia, we performed immunolabeling against OLIG2 (oligodendrocytes), GFAP (astrocytes), and NeuN (neurons). The transplantations resulted in a varying number of integrated GFP-positive cells as determined by immunostaining for GFP. The majority of the transplanted control astroglia (LV-UbiC:GFP-transduced) yielded in GFAP-positive astrocytes (Figure 8A) while a minor fraction of transplanted cells formed oligodendrocytes (Figure 8B). We also observed a rather small fraction of transplanted LV-UbiC:GFP-transduced glia formed neurons with extended processes (Figure 8C). When LV-UbiC:STAT6VT-GFP-transduced astroglia were transplanted, we found that the transplanted cells did not form extended processes and the number of GFP-positive cells were significantly lower than the control transplantations and they displayed round, fragmented morphology with no processes (Figure 8D). A rather small minority of the UbiC:STAT6VT-GFP-transduced cells expressed oligodendrocyte marker OLIG2 (Figure 8E) and GFAP (Figure 8F), while no neurons were observed. This pattern was consistent in APP/PS1dE9 mouse brains where the difference in transplantation efficiency was apparent between transplanted control astroglia (Figure 8G, LV-UbiC:GFP-transduced) and STAT6VT-expressing astroglia (Figure 8H, LV-UbiC:STAT6VT-GFP-transduced). Altogether, these results suggest that expression of IL4R or STAT6VT in astroglia impairs the survival of these cells *in vivo* but not *in vitro*.

**FIGURE 9:**
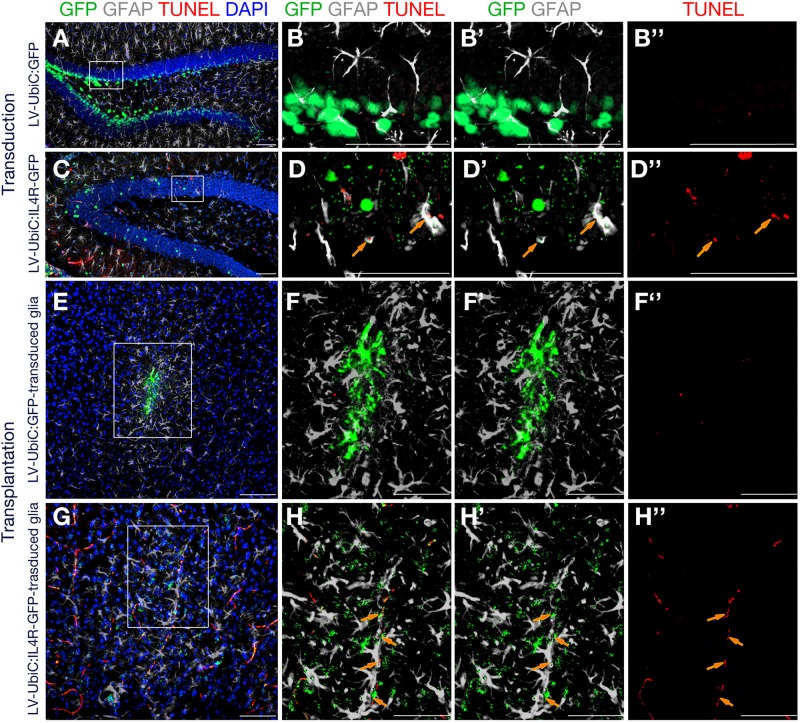
Immunostaining for GFAP (gray) and GFP (green) coupled to TUNEL staining (red). **(A)** Wild type mouse hippocampus transduced with Lv-UbiC:GFP. **(B)** Higher magnification of the framed region in **(A)** without DAPI. **(B′)** Overlaid GFP and GFAP channels. **(B′′)** TUNEL staining as single fluorescence channel. **(C)** Wild type mouse hippocampus transduced with Lv-UbiC:IL4R-GFP. **(D)** Higher magnification of the framed region in **(D)** without DAPI. **(D′)** Overlaid GFP and GFAP channels. **(D′′)** TUNEL staining as single fluorescence channel. **(E)** Wild type mouse cortex transplanted with Lv-UbiC:GFP-transduced astroglia. **(F)** Higher magnification of the framed region in **(E)** without DAPI. **(F′)** Overlaid GFP and GFAP channels. **(F′′)** TUNEL staining as single fluorescence channel. **(G)** Wild type mouse cortex transplanted with Lv-UbiC:IL4R-GFP-transduced astroglia. **(H)** Higher magnification of the framed region in **(G)** without DAPI. **(H′)** Overlaid GFP and GFAP channels. **(H′′)** TUNEL staining as single fluorescence channel. Orange arrows show TUNEL-positive, transduced glia. All animals are WT. *n* ≥ 3 wild type animals. Scale bars: 100 μm.

To test our hypothesis, we performed TUNEL staining to detect apoptotic cells after direct transduction of LV-UbiC:GFP or LV-UbiC:IL4R-GFP or transplantation of transduced astroglia in wild type animals (Figure 9). Unlike the transduction with LV-UbiC:GFP (Figures 9A–B′′), transduction with LV-UbiC:IL4R-GFP resulted in TUNEL-positive astroglia (Figures 9C—D′′). Similarly, while the transplantation of LV-UbiC:GFP-transduced astroglia did not lead to TUNEL-positive glia (Figures 9E–F′′), LV-UbiC:IL4R-GFP-transduced astroglia displayed TUNEL reactivity (Figures 9G–H′′). These results support our findings that expression of IL4R in astroglia leads to cell death.

## Discussion

The restoration of neural tissue is of clinical importance especially in neurodegenerative diseases. However, in mammals, the neurogenic ability declines with aging and the pool of the stem cells decreases (Ekdahl et al., 2003; Monje et al., 2003; Borsini et al., 2015). Additionally, during disease progression, neural stem cell proliferation and neurogenesis reduce even further. In AD patients, neurogenesis reduces dramatically and the decrease in adult neurogenesis could be a contributing factor to the pathology (Tincer et al., 2016; Hollands et al., 2017; Kizil and Bhattarai, 2018; Teixeira et al., 2018; Choi and Tanzi, 2019; Moreno-Jimenez et al., 2019; Cosacak et al., 2020). Therefore, studying regenerating organisms such as the zebrafish to learn how to counteract the pathology-suppressed neurogenic ability, and neural stem cell plasticity could be a promising approach to develop circuit resilience and brain repair in AD. Based on our previous findings (Bhattarai et al., 2016; Cosacak et al., 2017, 2019; Papadimitriou et al., 2018), we proposed that IL4 could be a factor to coax mammalian astroglia *in vivo* to become proliferative and neurogenic in disease conditions.

In our previous work, we found that zebrafish uses IL4 signaling as crosstalk to activate glial cells toward proliferation and neurogenesis (Bhattarai et al., 2016). In 3D cultures of human neural stem cells and astroglia, IL4 receptor (IL4R) is expressed and IL4 can revert the AD-associated reduction of astroglia proliferation and neurogenesis (Papadimitriou et al., 2018). These results suggested that IL4 signaling could be used to enhance neurogenesis and proliferation of astrocytes in a cell-autonomous manner by activating this signaling in astroglia. In the current study, we found that type 1 IL4/STAT6 signaling obliterates astroglia *in vivo* in mouse brains. This correlates with previous findings that IL4 could promote apoptosis through a caspase-dependent mechanism in microglia (Soria et al., 2011). IL4 was reported to promote differentiation, proliferation, and survival of different tumor cells through its interaction with IL4R (Koller et al., 2010; Venmar et al., 2014; Kim et al., 2016). For instance *IL4R* is a biomarker for various aggressive forms of glioblastoma multiforme (Puri et al., 1994; Joshi et al., 2001; Scheurer et al., 2008; Gadani et al., 2012). Additionally, glioblastoma cells would evade apoptosis in correlation with *Il4r* expression and enhance growth unlike healthy astroglia (Barna et al., 1995; Debinski, 1998). This suggests that type 1 IL4 signaling in glia induces apoptosis unless a neoplastic transformation evades apoptosis and allows IL4 signaling to promote proliferation of astroglia, which happens *in vivo* in zebrafish and *in vitro* in mammalian cells. It should also be noted that the effects of type 1 IL4R signaling on astroglia might be non-cell autonomous as many cells express the receptor after transduction. Additionally, the non-permissive environment hypothesis can include the effects of other cells in the brain on IL4R-expressing astroglia. Further research is needed to clarify this aspect.

We suggest that evolutionarily, mammalian brains developed a non-permissive environment for astroglia that have active IL4 signaling for its potential effects on hyper-proliferation. Under apoptosis-evading conditions of tumors, IL4 receptor (IL4R) is promoting proliferation and blockage or hypomorphic nucleotide polymorphisms in IL4R reduce the aggressiveness of glial tumors (Scheurer et al., 2008). Therefore, our work suggests that an evolutionary divergent role for signaling pathways (such as the IL4 signaling) in astroglia might underlie the disparity between the proliferative and neurogenic properties of mouse and zebrafish astroglia in health and disease. This difference might have functional ramifications in the regenerative outputs of zebrafish and mouse brains.

We would like to note that investigating complex diseases of humans in non-human model organisms is challenging. Recapitulation of the pathological culprits of a disease faithful to the human pathology is unlikely to fully succeed in a model due to inherent physiological differences between the cells of humans and other organisms, even rodents (Qiu et al., 2016; Hodge et al., 2019). However, reductionist models of human diseases in appropriate organisms are quite powerful for addressing particular aspects of pathologies or for designing experimental treatment options that may defy that particular disease. For instance, zebrafish proposed many signaling pathways that could be harnessed for enhanced responses in tissue counterparts in humans (Zon, 1999; Tomasiewicz et al., 2002; Poss et al., 2003; Rubinstein, 2003; Lieschke and Currie, 2007; Newman et al., 2010; Diep et al., 2011; Kizil et al., 2012a, b; Kyritsis et al., 2012; Gemberling et al., 2013; MacRae and Peterson, 2015; Mokalled et al., 2016; Papadimitriou et al., 2018; Celikkaya et al., 2019; Cosacak et al., 2019; Reinhardt et al., 2019). Other disease models in zebrafish yielded in useful information on the pathological mechanisms and led to the development of promising drugs (Cully, 2019).

A peculiarity of zebrafish that is appealing to us is its regenerative ability. Provided that the molecular basis of regenerative neurogenesis is understood in the zebrafish brain, we may have the chance to pinpoint what is missing in mammalian brains and how this “gap” can be filled. Our current study counts among the first such comparative approaches, which we believe will become a norm and will flourish as the reliability of zebrafish disease models prove to be of high relevance to humans. We also emphasize that as a general comparative analysis pipeline, using the experimental data acquired from the disease models; humanized models such as 3D cell cultures can be employed for the validity of the findings in human cells. Finally, the differences in the inherent complexity and the cellular physiology of mammalian brains may render such comparative analysis challenging and the findings in zebrafish may not be directly applicable to mammals in some cases. Such incongruences will also enhance our understanding from an evolutionary standpoint as to why and how mammalian brains lost their regenerative power, and which cell types and in what specific context must be nudged to become regenerative. This audacious workflow will surely increase the comparative power of the findings in zebrafish and validate the reliability of the use of this model in AD research.

## Data Availability Statement

The datasets generated for this study can be found in the GEO GSE140793.

## Ethics Statement

The animal study was reviewed and approved by Landesdirektion Sachsen, Germany. Permit number: TVV 87/2016.

## Author Contributions

VM and CK conceived and designed the experiments and wrote the manuscript. VM performed the experiments and acquired the data. AD, SR, and AP contributed to the generation of sequencing data. PB, TS, KB, NG, and RL contributed to the experimental procedures or provided samples. SP prepared virus particles. MC analyzed the single cell sequencing data. VM, TS, PB, SP, NG, RL, and CK edited the manuscript.

## Conflict of Interest

The authors declare that the research was conducted in the absence of any commercial or financial relationships that could be construed as a potential conflict of interest.

## Footnotes

1 https://www.ncbi.nlm.nih.gov/geo

## Supplementary Material

FIGURE S1 Lentiviral backbone that is used for generating Lv constructs for IL4R and STAT6VT. Genes of interests were cloned into upstream region in frame to EGFP.

FIGURE S2 **(A)** Schematic workflow for testing neurogenicity of dentate gyrus progenitors in culture. **(B)** Immunostaining for TUBB3 and GFAP at 1 week after growth factor withdrawal. **(C)** Immunostaining for TUBB3 and GFAP at 2 weeks after growth factor withdrawal. **(D)** Quantification charts indicating the relative abundance of GFAP-positive astroglia and TUBB3 positive neurons.
