# Supplementary figures and images for: Type 1 Interleukin-4 Signaling Obliterates Mouse Astroglia in vivo but Not in vitro
Source: Front Cell Dev Biol. 2020 Feb 26;8:114. doi: 10.3389/fcell.2020.00114 (PMC7057913; doi:10.3389/fcell.2020.00114)

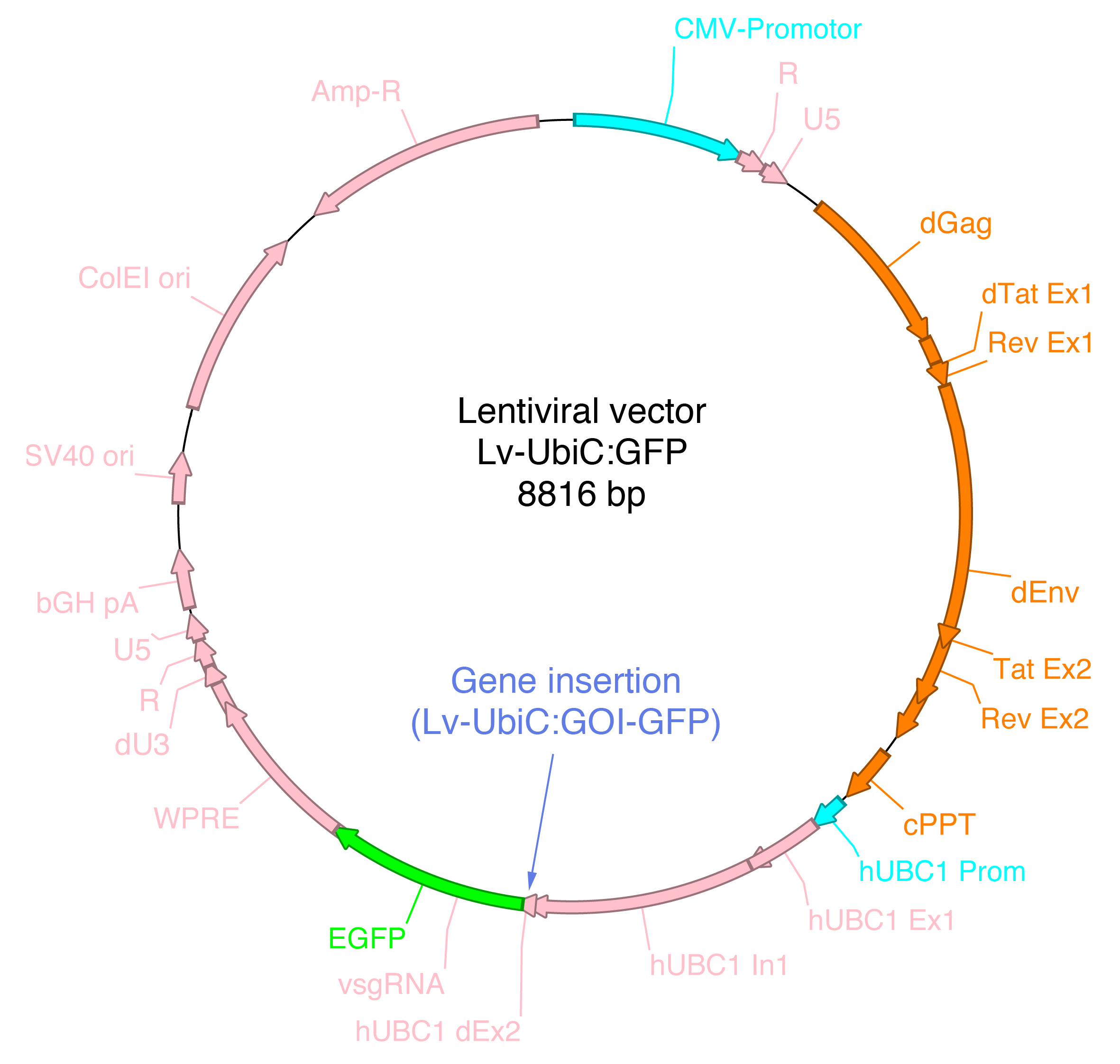

Supplement: FIGURE S1 — Lentiviral backbone that is used for generating Lv constructs for IL4R and STAT6VT. Genes of interests were cloned into upstream region in frame to EGFP. [file Image_1.JPEG]

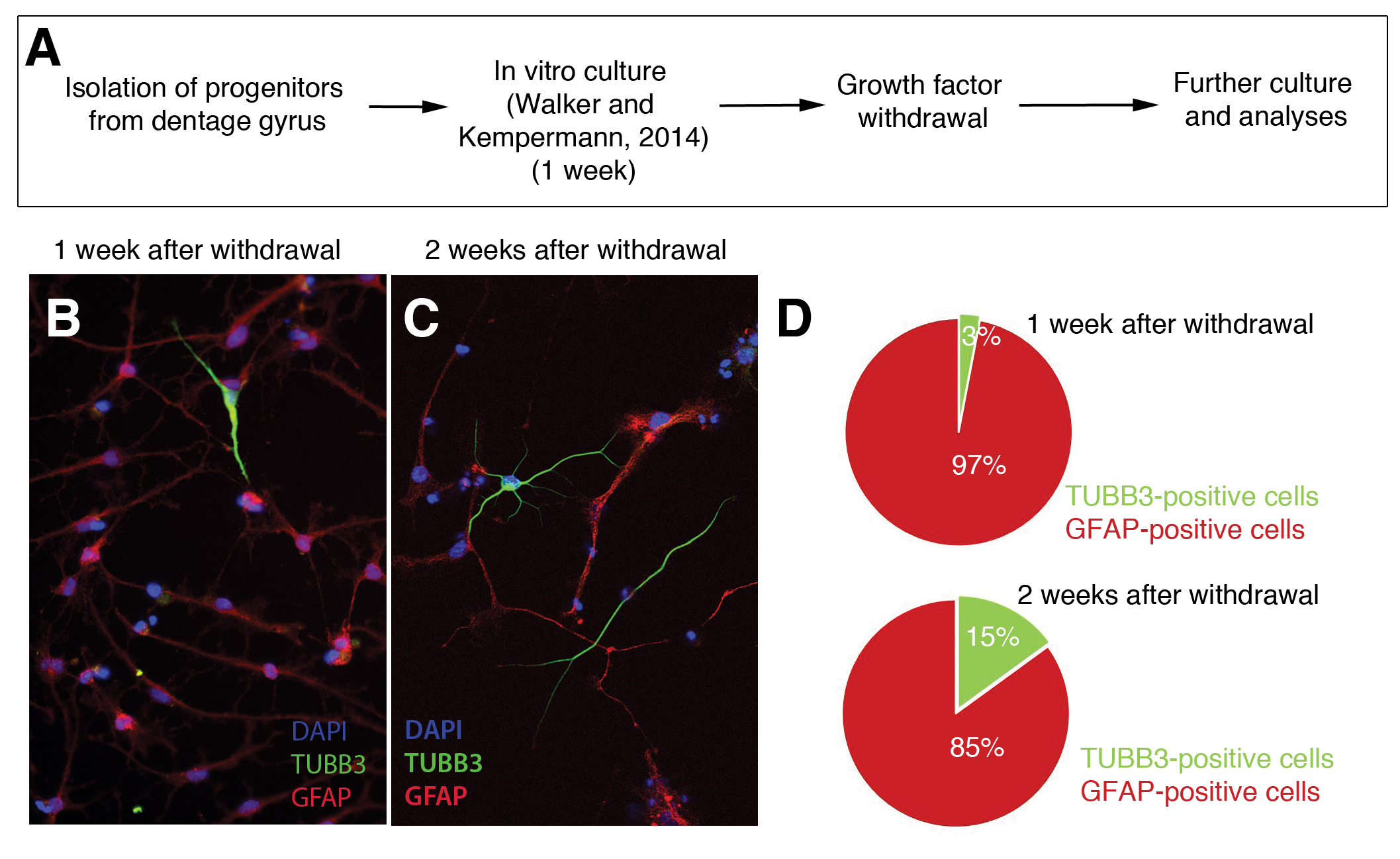

Supplement: FIGURE S2 — (A) Schematic workflow for testing neurogenicity of dentate gyrus progenitors in culture. (B) Immunostaining for TUBB3 and GFAP at 1 week after growth factor withdrawal. (C) Immunostaining for TUBB3 and GFAP at 2 weeks after growth factor withdrawal. (D) Quantification charts indicating the relative abundance of GFAP-positive astroglia and TUBB3 positive neurons. [file Image_2.JPEG]
